# The Effect of Medical Therapies for Subthreshold Abdominal Aortic Aneurysm Growth and Mortality: A Network Meta-Analysis of Randomized Controlled Trials

**DOI:** 10.1093/icvts/ivag088

**Published:** 2026-03-24

**Authors:** Phil Yi Jun Lu, Ishith Seth, Casey Hiu Ching Fung, Ramon Varcoe, Warren Rozen, Konrad Joseph

**Affiliations:** Vascular Surgery Unit, Department of Surgery, Frankston Hospital, Peninsula Health, Melbourne, VIC 3199, Australia; Vascular Surgery, Department of Surgery, Alfred Health, Melbourne, VIC 3004, Australia; Plastic Surgery Unit, Department of Surgery, Frankston Hospital, Peninsula Health, Melbourne, VIC 3199, Australia; Cardiothoracic Surgery, Austin Health, Melbourne, VIC 3084, Australia; Vascular Surgery, Prince of Wales Hospital, Sydney, NSW 2031, Australia; Plastic Surgery Unit, Department of Surgery, Frankston Hospital, Peninsula Health, Melbourne, VIC 3199, Australia; Department of Surgery, Port Macquarie Base Hospital, Port Macquarie, NSW 2444, Australia

**Keywords:** Abdominal aortic aneurysm, AAA, medical therapy management, mortality, network meta-analysis

## Abstract

**Objectives:**

Abdominal aortic aneurysm (AAA) is often fatal when ruptured, and current guidelines suggest surgical management at suprathreshold sizes (50 mm for women or 55 mm for men) or with rapid expansion (>5 mm/year). Many medical therapies have been assessed for reducing subthreshold AAA expansion, though the evidence remains inconclusive. This network meta-analysis (NMA) compares AAA growth and mortality amongst medical treatments for AAAs.

**Methods:**

MEDLINE (via PubMed), Scopus, Web of Science, EBSCO, and Cochrane Library databases were searched for relevant randomized controlled trials (RCTs) from database inception to 2024. Outcomes assessed included AAA growth rate, rate of referral for aneurysm surgery, overall mortality, and discontinuation from adverse effects. Data were analysed using R software, and *P*-score were used to rank different treatments. The GRADE framework was performed to assess the quality of evidence.

**Results:**

Thirteen RCTs comprising 3084 patients were included in this NMA. Abdominal aortic aneurysm diameters ranged from 3.1 to 4.6 cm in the intervention group and 3.5-4.5 cm in the placebo group. Study-level mean annual growth rate ranged from 1.2 to 2.8 mm/year in the intervention group compared with placebo (1.2-2.6 mm/year). There were no significant differences in AAA growth among the compared groups (*P*-score probability in brackets): propranolol (0.73), telmisartan (0.66), antibiotics (0.53), placebo (0.53), ACE inhibitors (0.52), ticagrelor (0.46), and pemirolast (0.06). There were no significant differences among the compared groups in terms of aneurysm surgery referral rates, with propranolol (0.91), antibiotics (0.56), placebo (0.45), and pemirolast (0.08) showing similar outcomes. Similarly, no significant differences were observed in overall mortality rates across the groups, including telmisartan (0.87), antibiotics (0.57), ACE inhibitors (0.51), placebo (0.35), and propranolol (0.17). However, propranolol (OR = 3.14, 95% CI, 1.34-7.35) and ticagrelor (OR = 5.10, 95% CI, 1.12-23.18) were associated with a higher rate of discontinuation due to adverse events. Most of the studies analysed demonstrated moderate quality evidence.

**Conclusions:**

Current evidence highlights ongoing uncertainty regarding the efficacy of medical therapies in reducing subthreshold AAA growth rates, rates of referral for surgical repair, or overall mortality. The absence of statistically significant benefit may reflect underpowered datasets rather than definitive treatment inefficacy. Future large-scale, appropriately powered RCTs evaluating emerging medical treatments are required to accurately assess their clinical potential.

## Graphical abstract

**Figure:**
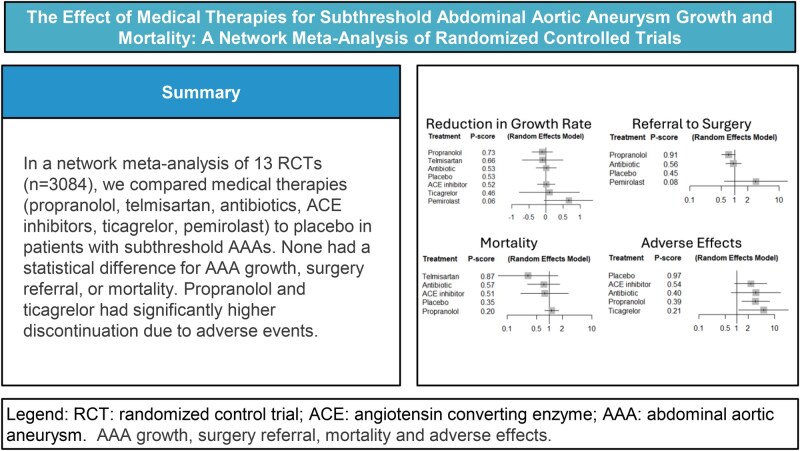

## INTRODUCTION

Abdominal aortic aneurysm (AAA) is defined as a dilation of the abdominal aorta, exceeding 1.5 times the normal diameter or an absolute threshold of 30 mm.^1^^,^^2^ It is a prevalent condition, particularly among older adults, with a multifactorial aetiology involving genetic predisposition, environmental factors, and vascular risk factors such as smoking, male sex, hypertension, and hyperlipidaemia.^1^^,^^2^ The pathophysiology of AAA is complex, characterized by chronic inflammation, oxidative stress, loss of elastic lamina with smooth muscle cellular apoptosis, and extracellular matrix degradation, with atherosclerosis and intraluminal thrombus, results in progressive weakening of the aortic wall and contributes to aneurysmal expansion^3^ with the eventual risk of rupture.

Rupture of an AAA is a catastrophic event with a mortality rate of 60% to 80%,^3^^,^^4^ emphasizing the need for timely identification and management. Current clinical guidelines from the European Society for Vascular Surgery (ESVS)^5^ and the Society for Vascular Surgery (SVS),^6^ recommend elective surgical repair when the aneurysm diameter reaches 55 mm in men and 50 mm in women, or when rapid expansion (>10 mm/year) is observed. These thresholds are based on the balance between the risk of rupture and the risks associated with surgical intervention. Preventing rupture remains the cornerstone of AAA management, with periodic surveillance for smaller aneurysms to monitor their growth. Ultrasound is the primary modality for initial diagnosis and ongoing surveillance due to its high sensitivity, specificity, and cost-effectiveness. Regular monitoring intervals are recommended based on aneurysm size and patient characteristics, which should consider life expectancy, suitability for repair, and individual preferences.^5^ Computed tomography angiography (CTA) is often employed for detailed anatomical assessment when surgical intervention is being considered.

Treatment options for threshold or rapidly expanding AAA include open surgical repair (OSR) and endovascular aneurysm repair (EVAR).^7^ While OSR remains the gold standard for younger patients with low operative risk, EVAR has gained popularity for its lower perioperative morbidity and faster recovery. Currently, EVAR remains the preferred method of treatment for ruptured aneurysms.^6^ For subthreshold AAAs (40-54 mm), it is thought that the risks of intervention are higher than the risk of rupture when considering procedural complications, mortality, and long-term device-related issues.^8^^,^^9^ Consequently, medical management has been investigated as an adjunctive strategy to reduce cardiovascular risk factors,^6^ delay aneurysm growth, and reduce the need for surgical intervention.

The medical management of subthreshold AAAs has gained attention as a potential strategy to slow aneurysmal growth and delay the need for surgical intervention. Despite this interest, current evidence on medical therapies for AAA is inconclusive. Various agents, including metformin, statins, and fluoroquinolones, have been investigated for their potential to mitigate the inflammatory and proteolytic pathways implicated in aneurysm progression. Metformin^10^ and statins^11^^,^^12^ have been shown in some studies to reduce the rate of growth and rupture risk of AAA, suggesting that there may be a role for medical management in subthreshold AAAs. However, the current evidence is inconclusive, and concerns regarding adverse effects have limited their widespread adoption. The lack of robust, high-quality evidence highlights the need for comprehensive analyses to clarify the efficacy and safety of these therapies.

A network meta-analysis (NMA) offers a powerful approach to address this gap by synthesizing evidence from direct and indirect comparisons across multiple interventions. By leveraging data exclusively from randomized controlled trials (RCTs) for this NMA, we provide a rigorous and comparative assessment of available medical therapies for subthreshold AAAs, allowing for ranking interventions based on their relative effectiveness and safety,^13^ thereby guiding clinical decision-making and informing future research directions. We aim to evaluate the effects of medical therapies on AAA growth, mortality, and medical treatment-related adverse events, with the goal of informing evidence-based practice and optimizing patient outcomes.

## MATERIALS AND METHODS

The current study was reported according to the preferred reporting items for systematic reviews incorporating network meta-analyses and the Cochrane Handbook of Systematic Reviews.^14^ As per the Cochrane Handbook of Systematic Reviews, ethics from the governing board were not required for a systematic review and NMA.

### Systematic search strategy

We systematically searched the MEDLINE (via PubMed), Scopus, Web of Science, EBSCO, and Cochrane Library of Clinical Trials electronic databases from 1901 to June 2024. All steps of this study were conducted in accordance with the Cochrane Handbook of Systematic Reviews of Interventions in addition to the Preferred Reporting Items for Systematic Reviews and Meta-Analyses (PRISMA) statement guidelines (attached in File SA). The search terms accessed individually or in combination included “abdominal aortic aneurysm,” “AAA,” “aortic aneurysm,” “medical therapy,” “pharmacological treatment,” “beta-blocker,” “propranolol,” “statin” (including “Atorvastatin,” “Rosuvastatin,” and “Pravastatin”), “angiotensin convertase enzyme (“ACE”) inhibitors,” “angiotensin-II receptor blockers” (“ARB”), “telmisartan,” “metformin,” “statins,” “antibiotic,” “azithromycin,” “roxithromycin,” “doxycycline,” “ticagrelor,” and “pemirolast.” Medical Subject Headings terms were used where applicable. Reference lists of included studies were manually reviewed for any additional eligible RCTs. This meta-analysis was listed on the PROSPERO International Prospective Register of Systematic Review (CRD42021284345).

### Study selection

Two investigators screened the title/abstract and full text of retrieved publications independently. A third reviewer resolved any disagreements. The studies’ inclusion criteria were restricted to RCTs published in English that investigated the efficacy of propranolol, ACE inhibitors, ARBs, antibiotics, ticagrelor, and pemirolast for the treatment of subthreshold AAAs (defined as those <49 mm and not yet planned for surgical repair). The exclusion criteria included observational studies, case reports, case series, reviews, editorials, commentaries, non-English studies or studies that were unable to translate into English and theses.

### Data collection

For each included RCT, data on study and patient characteristics were extracted by two reviewers independently using a data collection form. A third reviewer resolved disagreements. Extracted study and patient characteristics included the first author’s name, year of publication, study design, number of patients in each group, mean age, male percentage, AAA diameter entry criteria (mm), method of AAA measurement, interventions, primary outcome, and follow-up duration. The following statistical outcomes were further extracted from each included study: AAA growth, referral to aneurysm surgery, overall mortality, and discontinuation owing to adverse events.

### Methodological and risk of bias assessment

The quality of included RCTs was assessed using the Risk of Bias Tool developed by the Cochrane Collaboration.^15^ For each RCT, a score of high, low, or unclear bias was assigned to each of the following items: sequence generation, allocation concealment, blinding of participants, personnel, and outcome assessors, incomplete outcome data, selective outcome reporting, and other potential sources of bias. Individual disagreements were resolved by consensus.

### Statistical analysis

All analyses were performed using R version 3.5.2 (R: A language and environment for statistical computing; R Foundation for Statistical Computing). Network meta-analysis using a frequentist approach was implemented through the “netmeta” statistical package (version 1.4-0) in R software.^16^ Continuous data were pooled as mean difference (MD) with a 95% CI, and dichotomous data were pooled as odds ratio (OR) with a 95% CI. Differences were considered statistically significant when the 95% CI did not include 0 for MD and 1 for OR. Heterogeneity was defined as the variability of results across studies using the Cochran’s Q-test, with *P*-value >.1 indicating no significant heterogeneity and *P*-value ≤.1 indicating significant heterogeneity. The random-effects model was used to provide a conservative estimate of the treatment effect. We used the net-split method to assess inconsistency between direct and indirect estimates and measured it by generalized Cochran’s Q statistics for multivariate meta-analysis as described by Krahn et al.^17^ The *P*-score was used to rank the treatments, with a larger value indicating better performance,^18^ but in the absence of a statistically significant result, it does not imply clinical superiority.

### Data availability

This study is a systematic review and meta-analysis of previously published data. No new primary data were generated. All included studies are cited in the reference list, and extracted data used in the analysis are available from the corresponding author upon reasonable request.

## RESULTS

### Search strategy

**Figure 1.**
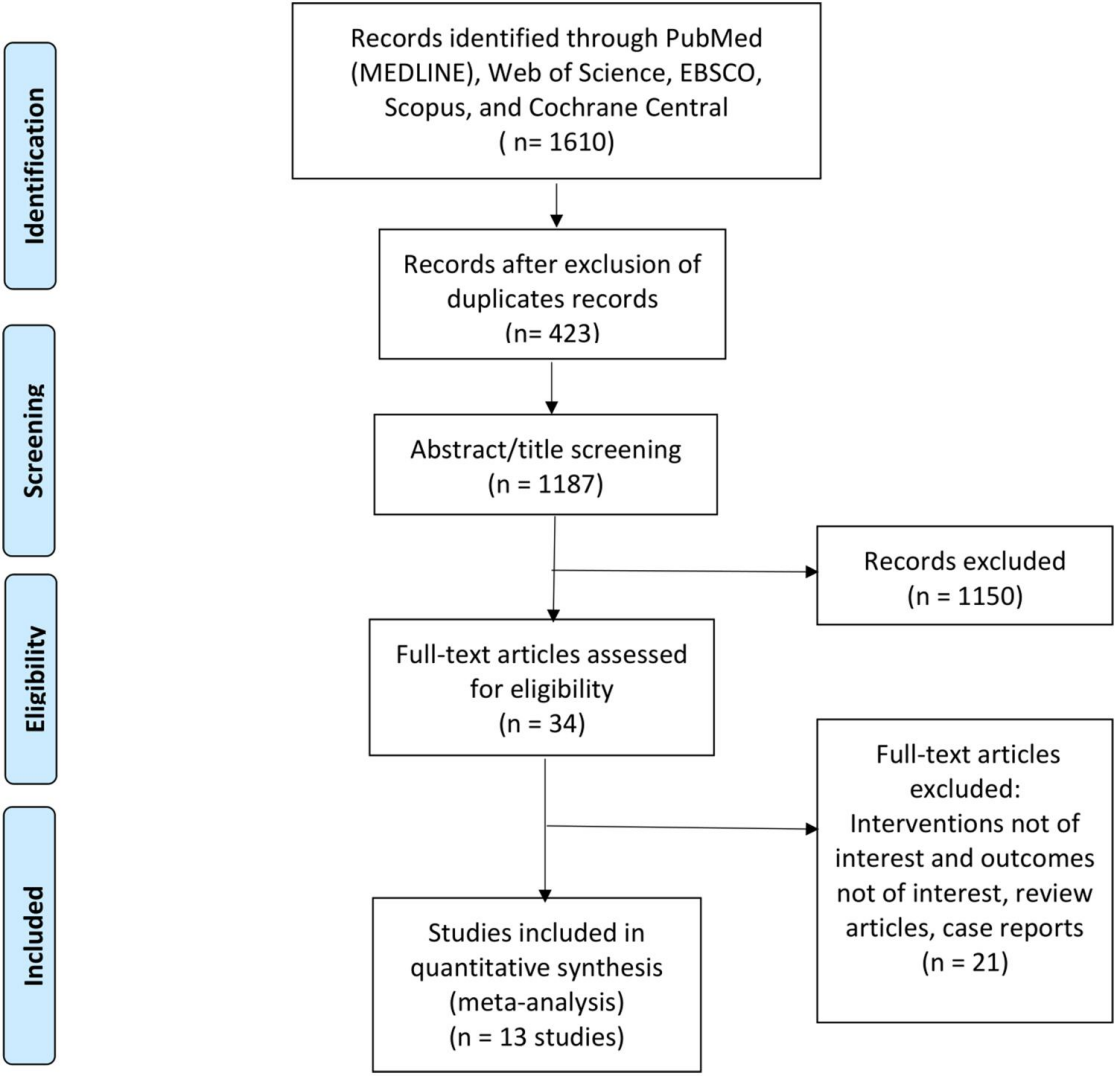
PRISMA Flow Diagram of Study Selection

A total of 1610 relevant papers were identified using the search strategy and imported into EndNote X9 software. A total of 423 duplicate papers were excluded, and a further 1150 papers were excluded from analysis after the title and abstract screening. Based on full-text screening, 13 papers were included for final review. The study selection process is shown in Figure 1.

### Baseline characteristics of included studies

Thirteen RCTs, with 3084 patients were included in the current study. RCTs assessed the following medications compared to placebo: propranolol,^19–21^ roxithromycin,^22^^,^^23^ azithromycin,^24^ doxycycline,^25–27^ pemirolast,^28^ ticagrelor,^29^ perindopril,^30^ and telmisartan.^31^ Metformin and statins were not included in the current study, as RCTs were identified in our search. The mean age of patients ranged from 68.8 to 72 years in the intervention group and from 68.1 to 73.8 years in the control group. Male patients accounted for 80% of the population included in this study. Reported AAA diameters ranged from 30 to 54 mm. Ultrasound, computed tomography (CT), and magnetic resonance imaging (MRI) were all methods used by studies to measure AAA diameter. Measurement was assessed for quality by a senior radiologist or ultrasonographer in all studies. The aneurysm growth rate was averaged per 1 year, from the sequential AAA diameter measurements over the time of follow-up.

**Figure 2.**
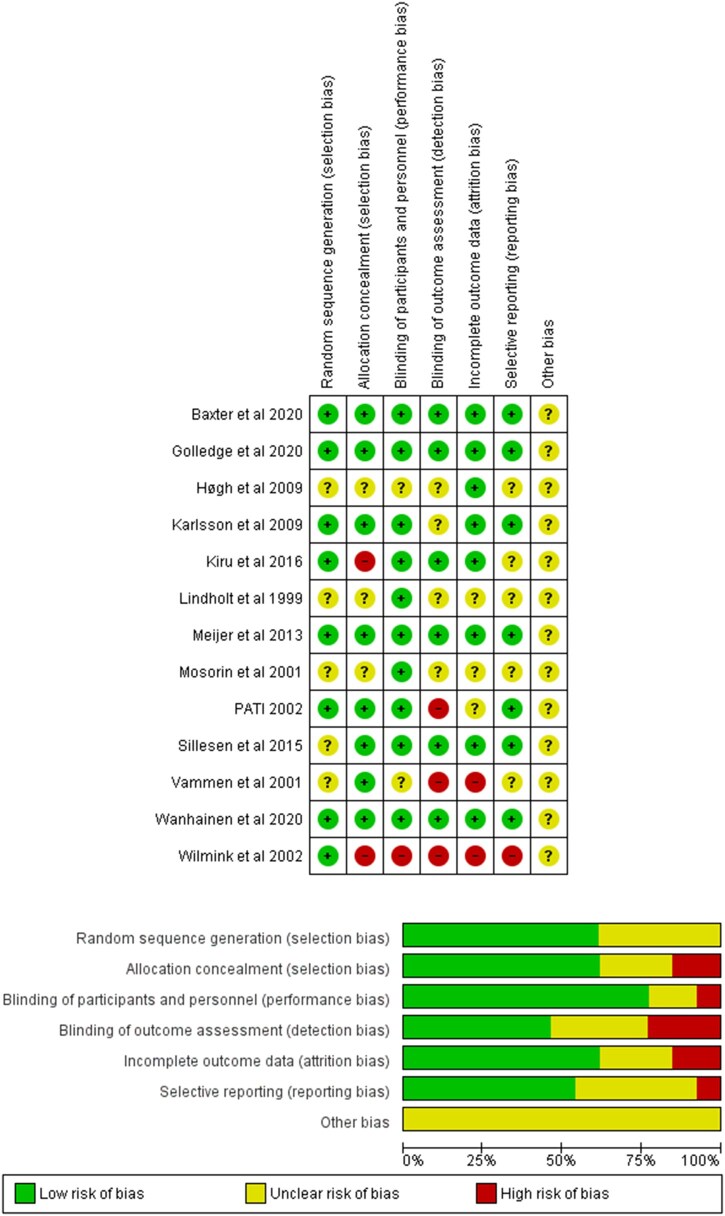
Summary of the Risk of Bias of Included Randomized Control Trials and Graph of Risk of Bias of Included Randomized Control Trials

The baseline characteristics of included studies are summarized in Table 1. The mean (SD) baseline maximum transverse diameter ranged from 31 to 46 mm in the intervention group and from 35 to 45 mm in the placebo group. The follow-up duration ranged from 18 to 24 months. The summary of the risk of bias evaluation of included RCTs was reported in Figure 2.

**Table 1.** Characteristics of Included Studies

Study	Study design	Number of patients	Mean age		Male		AAA diameter entry criteria (mm)	Method of AAA measurement	Treatment	Primary outcome	Follow-up (months)
			Intervention	Control	Intervention	Control					
Baxter et al. 2020^25^	RCT	261	71.0 (7.5)	70.9 (7.3)	111 (86)	108 (86)	35–50	CT	Doxycycline 100 mg orally twice daily for 2 years	AAA growth	24
Golledge et al. 2020^31^	RCT	300	73.2 (8.1)	73.8 (7.8)	92 (87)	91 (90)	35–49	CT and ultrasound	Telmisartan 40 mg	AAA growth	24
Wanhainen et al. 2020^29^	RCT	144	69.5 (4.5)	68.3 (4.2)	65 (94.2)	68 (97.1)	35–49	MRI and ultrasound	Ticagrelor (90 mg twice daily) or identical placebo	AAA growth	12
Kiru et al. 2016^32^	RCT	227	71.6 (6.9)	70.7 (7.5)	71 (97)	74 (94)	30–54	Ultrasound	Perindopril (10 mg), amlodipine (5 mg) or placebo	AAA growth	24
Sillesen et al. 2015^28^	RCT	326	70⋅8 (6⋅2)		210 (73)	77 (92)	39–49	Ultrasound	Pemirolast 10 mg twice daily, 25 mg twice daily, 40 mg twice daily or matching placebo twice daily for 52 weeks	AAA growth	12
Meijer et al. 2013^27^	RCT	286	70 (7)	70 (8)	120 (83)	114 (80)	35–50 or ≥50 mm if unfit or declined repair	Ultrasound	Doxycycline 100 mg or identical placebo	AAA growth	18
Høgh et al. 2009^22^	RCT	84	71 (4.1)	71 (3.7)	84 (100)	0 (0)	30–50	Ultrasound	Roxithromycin 300 mg once daily or matching placebo for 28 days annually	NR	24
Karlsson et al. 2009^24^	RCT	247	71 (IQR: 67 to 74)	71 (IQR: 67 to 76)	84 (34)	79 (32)	35–49	Ultrasound	Azithromycin 600 mg once daily for 3 days, then 600 mg once weekly for 15 weeks or identical placebo similarly administered	AAA growth	18
PAT 2002^20^	RCT	552	69.1 (8.1)	68.7 (7.6)	227 (41)	233 (42)	30–50	Ultrasound	Propranolol or identical placebo aiming for 120 mg twice daily if tolerated	AAA growth	NR
Wilmink et al. 2002^19^	RCT	477	68.8 (8.5)	70.6 (8.3)	495 (100)	0 (0)	30–45	NR	Propranolol 40 mg daily	AAA growth	NR
Vammen et al. 2001^23^	RCT	92	72 (3.7)	73 (3.7)	92 (100)	0 (0)	≥30	Ultrasound	Roxithromycin 300 mg once daily or matching placebo for 28 days	AAA growth	24
Mosorin et al. 2001^26^	RCT	34	68.6 (64.4 to 71.3)	68.1 (64.1 to 73.0)	16 (47)	13 (38)	30–54	Ultrasound	Doxycycline 150 mg once daily or placebo for 3 months	NR	18
Lindholt et al. 1999^21^	RCT	54	68.7 (6.4)	69.6 (4.7)	54 (100)	0 (0)	30–49	Ultrasound	Propranolol 40 mg twice daily or placebo twice daily	NR	NR

Abbreviations: AAA = abdominal aortic aneurysm; NR=not reported; RCT = randomized controlled trials.

### Outcomes

No significant differences were observed among the interventions. The probability ranking *P*-scores do not imply clinical superiority in the absence of a statistically significant difference.

#### Abdominal aortic aneurysm growth

**Figure 3.**
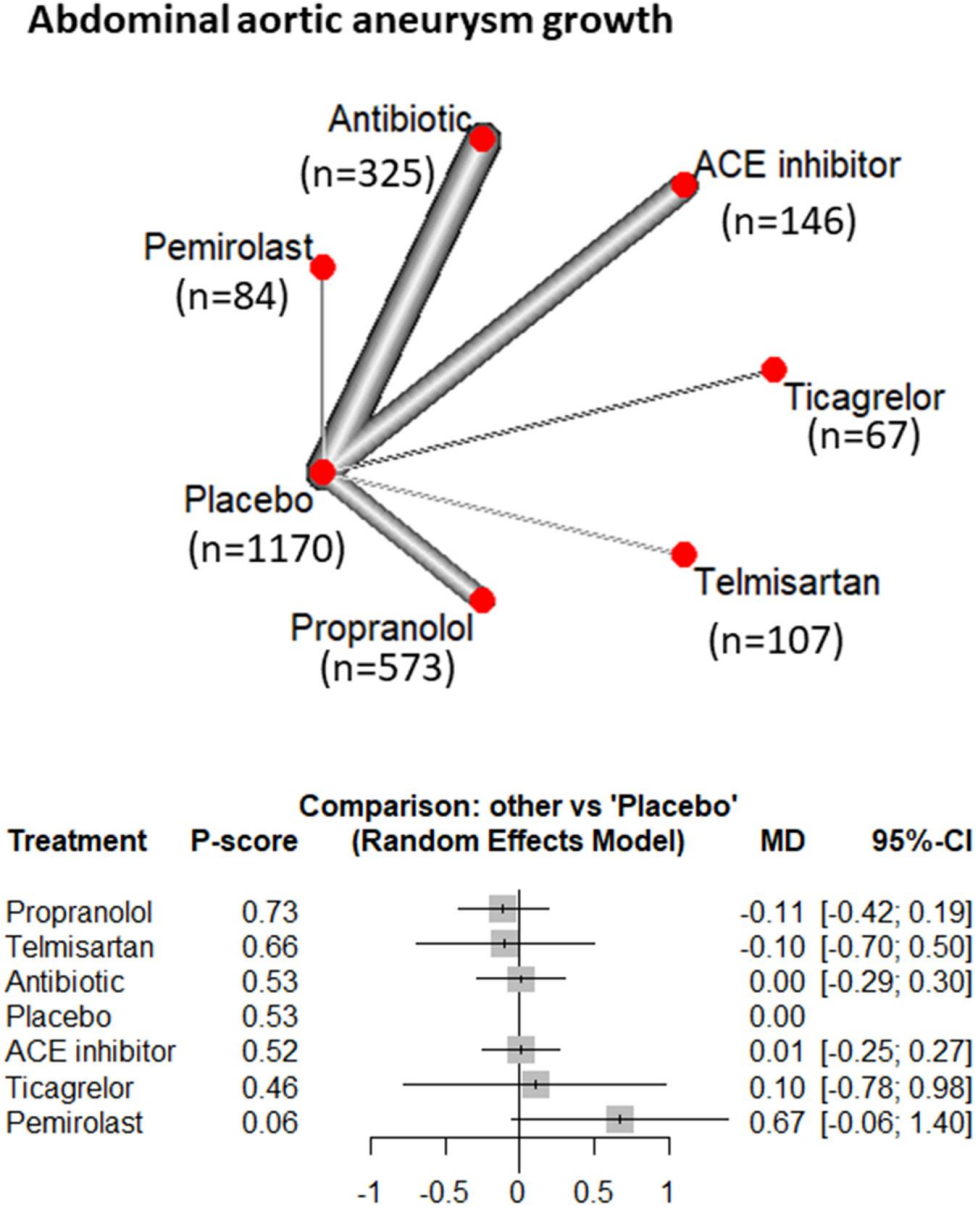
Network Meta-Analysis of Different Treatments Versus Placebo for AAA Growth, and Network Plot of AAA Growth Showing Direct Evidence among the Compared Treatments. The plot uses circles to represent each treatment and lines to connect treatments to their associated side effects. The size of each circle represents how many people were studied for that treatment, and the thickness of each line represents how many studies looked at that connection

Eleven studies, including 2421 patients and 7 interventions, were included in the NMA to evaluate AAA growth. The growth rate in diameter was reported in 8 RCTs as millimetres per year (mm/year). Across the included trials, the reported study-level mean annual growth rates ranged from 1.2 to 2.8 mm/year in the intervention groups and 1.2 to 2.6 mm/year in the placebo groups. No significant differences were observed among the interventions. The probability ranking *P*-scores are as follows: propranolol (0.73), telmisartan (0.66), antibiotics (0.53), placebo (0.53), ACE inhibitors (0.52), ticagrelor (0.46), and pemirolast (0.06). (Figure 3 and Table S1).

#### Referral to AAA surgery

**Figure 4.**
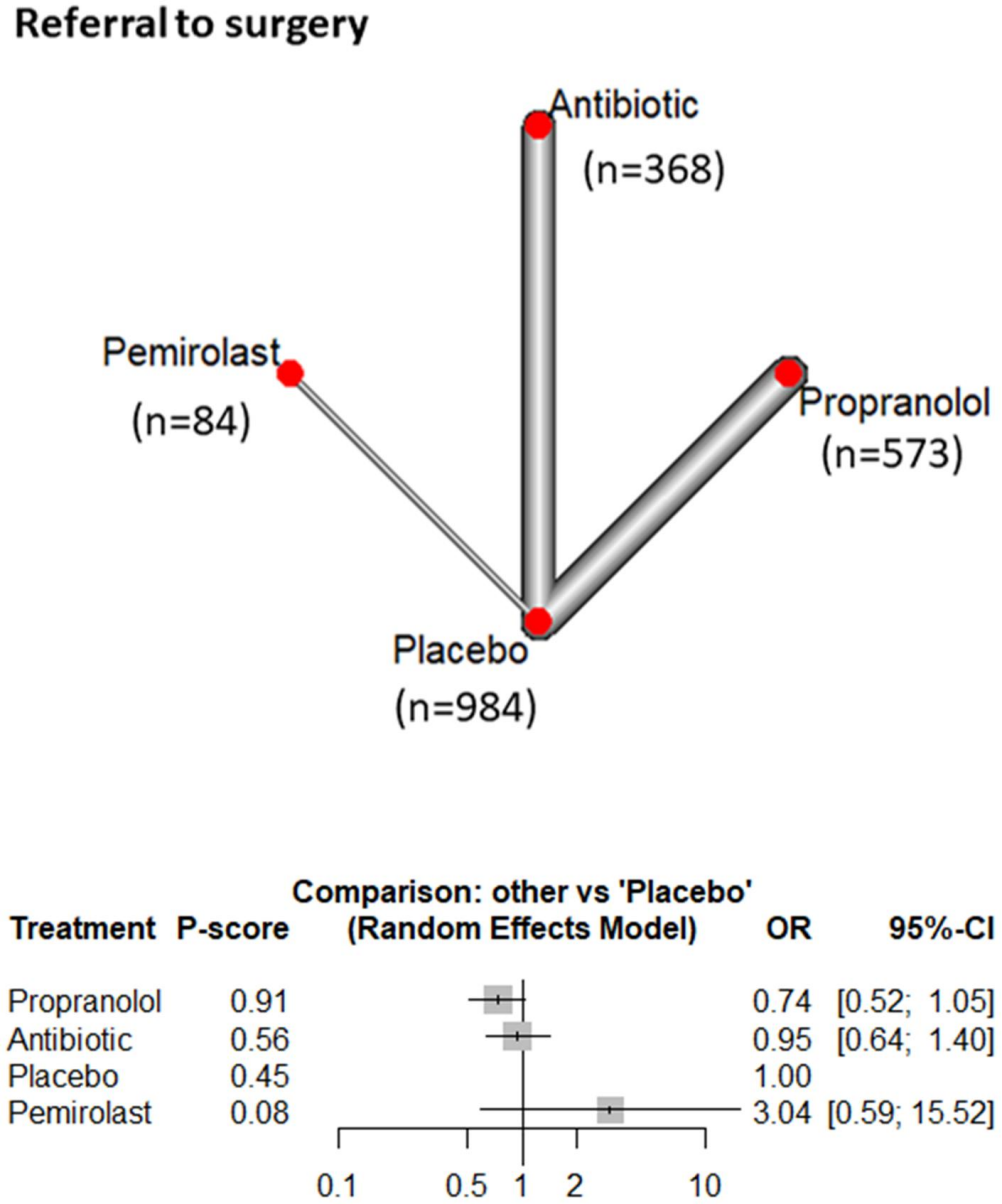
Network Meta-Analysis of Different Treatments Versus Placebo for Referral to Surgery, and Network Plot of Referral to Surgery Showing Direct Evidence among the Compared Treatments. The plot uses circles to represent each treatment and lines to connect treatments to their associated side effects. The size of each circle represents how many people were studied for that treatment, and the thickness of each line represents how many studies looked at that connection

Nine studies, with a total of 2009 patients, were included in the NMA to evaluate the effects of 4 interventions: propranolol, antibiotics, placebo, and pemirolast for the incidence of referral to AAA surgery. None of the interventions demonstrated a statistically significant reduction in referral for consideration of surgery. The associated *P*-scores were used to rank the probability of reduction in referrals: propranolol (0.91), antibiotics (0.56), placebo (0.45), and pemirolast (0.08) (Figure 4 and Table S2).

#### Overall mortality

**Figure 5.**
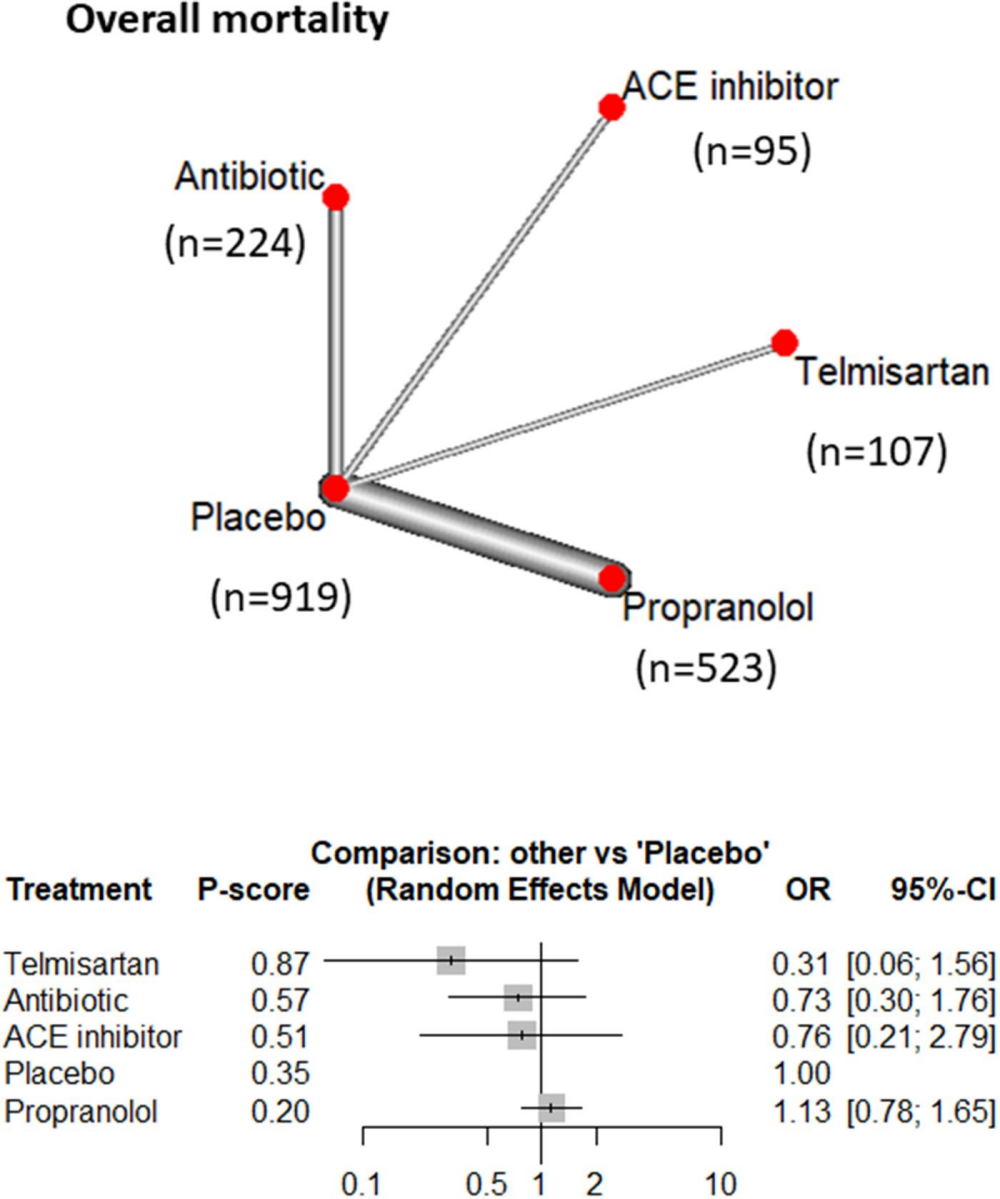
Network Meta-Analysis of Different Treatments Versus Placebo for Mortality, and Network Plot of Overall Mortality Showing Direct Evidence Among the Compared Treatments. The plot uses circles to represent each treatment and lines to connect treatments to their associated side effects. The size of each circle represents how many people were studied for that treatment, and the thickness of each line represents how many studies looked at that connection

The mortality rates reported in the included studies ranged from 0% to 20% across the follow-up periods, with most deaths not attributed to aneurysmal causes. The NMA for overall mortality included 9 studies, encompassing 1918 participants, and compared 5 intervention groups. The probability ranking *P*-score in mortality reduction were as follows: telmisartan (0.87), antibiotics (0.57), ACE inhibitors (0.51), placebo (0.35), and propranolol (0.17). However, none of the differences were statistically significant (Figure 5 and Table S3).

#### Adverse effects: all-cause discontinuation of medication

**Figure 6.**
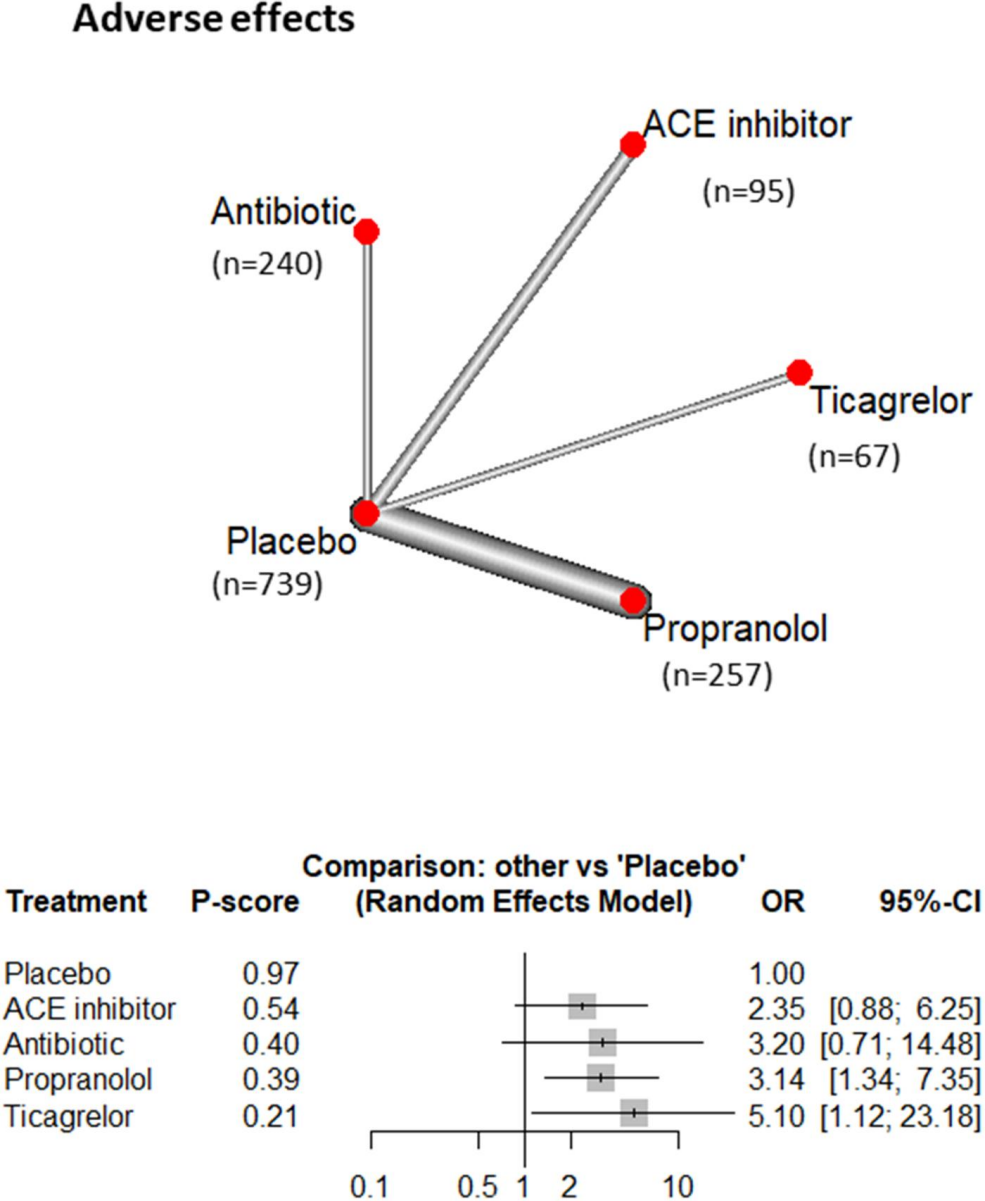
Network Meta-Analysis of Different Treatments Versus Placebo for Side Effects Requiring Cessation of Treatment, and Network Plot of Side Effects Showing Direct Evidence among the Compared Treatments. The plot uses circles to represent each treatment and lines to connect treatments to their associated side effects. The size of each circle represents how many people were studied for that treatment, and the thickness of each line represents how many studies looked at that connection

The NMA for adverse effects, including fatigue, bronchospasm, heart failure, bleeding, and dyspnoea, encompassed 6 studies with a total of 1448 participants across 5 intervention groups. The analysis identified significantly higher rates of treatment discontinuation due to adverse events for propranolol (OR = 3.14, 95% CI, 1.34-7.35) and ticagrelor (OR = 5.10, 95% CI, 1.12-23.18). No other statistically significant differences were observed. The *P*-scores least associated with adverse effects were placebo (0.97), followed by ACE inhibitors (0.54), antibiotics (0.40), propranolol (0.39), and ticagrelor (0.21) (Figure 6 and Table S4).

### Heterogeneity and inconsistency

No significant heterogeneity was observed for AAA growth (*P* = .11; *I*^2^ = 36.1%), overall mortality (*P* = .56; *I*^2^ = 0%), or referral to aortic aneurysm surgery (*P* = .89; *I*^2^ = 0%). However, significant heterogeneity was detected among the included studies regarding therapy discontinuation due to adverse events (*P* = .02, *I*^2^ = 62.7%). Node-splitting analysis comparing direct and indirect estimates revealed no significant inconsistency across all included outcomes (*P* > .1).

## DISCUSSION

In this NMA of 13 RCTs comprising 3084 patients, currently investigated medical therapies were not associated with meaningful reductions in AAA growth, referral for aneurysm repair, or overall mortality. These findings are consistent with the literature^33^^,^^34^ and reflect the predominantly neutral effects observed across studies, in which annual aneurysm growth rates were largely indistinguishable between treatment and placebo groups. The absence of demonstrable benefit may be attributable to several factors, including small sample sizes, methodological heterogeneity, short follow-up durations, and the focus on subthreshold AAAs, which together limit statistical power to detect modest treatment effects. In addition, most evaluated agents were repurposed cardiovascular therapies rather than drugs specifically targeting AAA pathobiology, and many trials controlled for cardiovascular risk factors (such as systolic blood pressure), which are key determinants of aneurysm expansion and may therefore attenuate observable treatment effects. Furthermore, the intrinsically progressive and structurally irreversible nature of AAAs may limit the efficacy of late pharmacological intervention. Accordingly, the apparent lack of therapeutic benefit observed in this NMA should be interpreted with caution given the lack of statistical power to detect modest differences; however, these findings provide important considerations to inform clinical practice and underscore the need for adequately powered, long-term RCTs evaluating emerging AAA-specific therapies.

### Specific medications

Among the 6 RCTs evaluating antibiotics for AAA management, short courses of roxithromycin (300 mg once daily for 28 days; *n* = 92)^23^ or doxycycline (150 mg once daily for 3 months; *n* = 34)^26^ were associated with significant reductions in aneurysm expansion compared with control groups. Furthermore, a 2009 trial by Høgh *et al.* (*n* = 84) reported that a 4-week course of roxithromycin reduced the subsequent need for AAA repair compared to placebo.^22^ However, these effects were not replicated in larger RCTs with a tapering azithromycin regimen over 15 weeks^24^ or prolonged doxycycline therapy (100 mg once or twice daily).^23^^,^^25^ In contrast, Meijer et al. observed accelerated AAA expansion during the first year among patients treated with doxycycline compared with placebo (2.8 vs 2.1 mm).^27^ Roxithromycin, a macrolide antibiotic with recognized anti-inflammatory and potential cardioprotective properties, has been explored as a candidate therapy for AAA.^23^ Proposed mechanisms include suppression of *Chlamydia pneumoniae* infection, which has been implicated in AAA pathogenesis, as well as broader anti-inflammatory effects that reduce atherosclerosis and hypertension.^23^ The development of *C. pneumoniae* targeted vaccination strategies has also been postulated as a potential approach to reducing AAA incidence and progression, although this remains theoretical and untested.^23^

Among the 5 RCTs evaluating antihypertensive therapies, propranolol was investigated in 3, telmisartan was tested in 1, and amlodipine and perindopril were both tested in 1 study.^19^^,^^20^^,^^30^^,^^31^ Despite hypertension being a well-established risk factor for AAA development and progression, none of these agents demonstrated a significant effect on the reduction of aneurysm expansion, aneurysm-related surgical outcomes, or rupture when compared with placebo. Notably, Kiru et al. was the sole trial to incorporate pre-treatment blood pressure parameters into its inclusion criteria. In this study, patients who were initially ineligible due to hypertension were permitted to achieve target blood pressures (systolic <150 mmHg) using an alternative class of antihypertensive medication prior to formal enrolment. They also found no difference in aneurysmal growth with an alternative antihypertensive. One possible explanation for this lack of observed benefit is that the fixed dosing strategies employed may have been insufficient to achieve optimal systolic blood pressure control. Notably, these trials utilized uniform dosing across study cohorts rather than individualized dose titration, which differs from routine clinical practice where antihypertensive therapy is titrated to therapeutic response. These fixed-dose regimens may have been insufficient to reach clinically therapeutic targets; 2 papers demonstrate a significant reduction in blood pressure, 2 do not and 1 does not report the difference. In addition, adherence issues were reported in trials involving beta-blocker therapy, which may have further limited the ability to accurately assess the effect of propranolol on AAA progression.

Sillesen et al.^28^ evaluated 3 once-daily doses of pemirolast (20 mg, 50 mg, and 80 mg), a mast cell inhibitor, to assess its effect on AAA progression. Over a 12-month follow-up period, none of the dosing regimens demonstrated a reduction in aneurysm growth or AAA-related events compared with control. The rationale for investigating mast cell inhibition was derived from previous data which suggested that mast cells are a key component of the aortic inflammatory infiltrate, where a mast cell deficiency or inhibition reduced AAA progression in rodent models.^35^ While prior evidence suggests that intraluminal thrombus formation and platelet activation contribute to AAA progression independently of primary dilatative mechanisms,^36^^,^^37^ ticagrelor did not demonstrate a significant reduction in aneurysm growth compared with placebo over a 12-month period.^29^^,^^38^ In addition, Pinchbeck et al.^38^ assessed the effect of fenofibrate on circulating biomarkers associated with AAA pathogenesis, including osteopontin. This study was based on previous rodent studies that suggested fenofibrate inhibited AAA development in 2 mouse models related to downregulating aortic osteopontin.^39^^,^^40^ Despite a reduction in serum triglyceride levels, fenofibrate had no significant effect on AAA growth or on the circulating concentrations of the biomarkers evaluated.^38^

We note that propranolol and ticagrelor were associated with higher rates of treatment discontinuation, largely attributable to adverse events. In a prior trial evaluating propranolol, approximately 40% of participants discontinued therapy, which may have contributed to suboptimal systolic blood pressure control and, consequently, accelerated AAA expansion.^20^ Reported adverse effects in the propranolol group included fatigue (*n* = 24), bronchospasm (*n* = 16), and symptoms consistent with heart failure (*n* = 7). Similarly, in the trial conducted by Wanhainen et al.,^29^ ticagrelor use was associated with bleeding events in 33% of patients and dyspnoea in 26%. The high rates of discontinuation and poor adherence observed with these agents limit the reliability of their estimated treatment effects in RCTs and reduce their clinical suitability for the broader AAA population, underscoring the need to identify alternative therapeutic targets with more favourable tolerability profiles.

Furthermore, it is important to clarify that the investigated medications in the included trials were not administered as true monotherapy. Rather, they were prescribed as adjuncts to the patients’ standard background cardiovascular regimens. While a standard exclusion criterion across these RCTs was the prior or concurrent use of the specific study medication or its pharmacological class, many of the primary studies did not adequately report whether these background medical therapies were evenly balanced between the intervention and placebo arms. The widespread use of concomitant cardiovascular medications—which themselves manage risk factors that are key determinants of aneurysm expansion—could act as a significant confounder, potentially attenuating the observable treatment effects of the trialled agents. Additionally, the reliance on aggregate trial data rather than patient-level data in this NMA precludes any robust evaluation of combination therapies or potential synergistic drug effects.

### Other medications

Diabetics have an observed lower AAA rupture risk, which is thought to be attributed to metformin use^41^^,^^42^ where a meta-analysis of cohort studies investigating metformin demonstrated an association with significantly limited AAA expansion and lower AAA rupture-related mortality.^10^ Statins have also been investigated, though results have been conflicting.^11^^,^^12^

While the findings of this study are consistent with previous observations, they should also prompt further investigation in light of emerging observational evidence supporting the potential role of metformin and statins in attenuating AAA progression and reducing rupture-related mortality. Notably, our systematic search identified insufficient RCT data for these agents, precluding their inclusion in this NMA. Nonetheless, a prior systematic review and meta-analysis reported that statin therapy was associated with reduced AAA growth, a lower risk of rupture (OR 0.63), and decreased peri-operative mortality (OR 0.55) compared with controls.^11^ In a large case–control study, Wemmelund et al.^43^ similarly demonstrated that statin use was associated with a reduced risk of ruptured AAA (adjusted OR 0.73, 95% CI, 0.61-0.86) and lower 30-day mortality (46.1% vs 59.3%). Additionally, a meta-analysis of 10 cohort studies found that metformin use was associated with reduced AAA expansion (MD 0.72 mm/year, 95% CI −1.08 to −0.37; *P* < .00001), as well as lower rates of AAA repair and rupture-related mortality (OR 0.80, 95% CI, 0.66-0.96; *P* = .02).^10^ Although these studies were case-control observational in design and therefore excluded from the present NMA, their consistent findings suggest that statins and metformin may represent promising therapeutic candidates for AAA management. Accordingly, ongoing and future RCTs evaluating the efficacy and safety of metformin in AAA populations warrant close attention. It is also important to acknowledge that several included studies reported substantial non-aortic-related mortality, including deaths attributable to cardiovascular disease and respiratory failure, as noted by Baxter et al.^25^ and Meijer et al.,^27^ which may further complicate interpretation of aneurysm-specific outcomes.

### Aneurysmal morphology and aetiology

An important consideration when interpreting these results is the underlying aetiology and morphology of the aneurysms within the pooled study population. The typical pathophysiology of AAA involves chronic inflammation, extracellular matrix degradation, and atherosclerosis. Consequently, we assume that the vast majority of patients included in these trials presented with standard degenerative or atherosclerotic AAAs. However, potential heterogeneity in aneurysm aetiology was not uniformly addressed across the analysed literature. Only 4 of the 13 included trials explicitly documented the exclusion of patients with non-atherosclerotic aetiologies, such as connective tissue disorders (eg, Marfan syndrome) or mycotic aneurysms. Furthermore, specific anatomical morphology was rarely delineated; notably, Mosorin et al.^26^ explicitly included a patient with a saccular aneurysm, whereas the remaining studies did not differentiate between typical fusiform and saccular morphologies. This lack of transparency is clinically relevant. Aneurysms driven by connective tissue disorders or infectious processes possess distinct pathophysiological mechanisms, exhibit different natural histories, and often warrant differing medical and surgical management strategies compared to standard atherosclerotic aneurysms. Similarly, saccular aneurysms are generally considered to harbour a higher risk of rupture at smaller diameters, potentially altering the risk-benefit considerations of conservative medical management.

### Potential effect modifiers

A critical assumption underlying any NMA is transitivity, which necessitates that the included trials are sufficiently similar across clinical and methodological variables that could act as effect modifiers. In this analysis, a qualitative assessment reveals several variations across the included studies that may influence the exchangeability assumption. First, the baseline AAA diameter entry criteria varied considerably across the trials; while several trials included smaller subthreshold aneurysms starting at 30 mm, others restricted enrolment to aneurysms starting at 35 or 39 mm. Given that baseline diameter is a strong, non-linear determinant of future expansion rates, these differing inclusion thresholds could introduce variability in the observed growth trajectories. Second, the demographic composition—particularly sex distribution—exhibited significant heterogeneity. Whilst there was a predominance of male participants, as a subset of the trials which analysed exclusively male cohorts (100% male), others included a substantial proportion of female participants, such as the PATI (approx. 41% male) and Karlsson et al.^24^ (approx. 33% male) trials. Third, there were methodological differences in the imaging modalities utilized to assess the primary outcome; the majority of studies relied solely on ultrasound (subject to significant inter-operator variability), whereas others incorporated CT or MRI, which may affect measurement precision. Finally, as these investigated agents were generally administered alongside differing standard cardiovascular background therapies (eg, statins and antiplatelets), unmeasured differences in baseline medical management across the network could potentially confound the results or attenuate the observable effects of the study drugs. Although sample size limitations precluded a formal meta-regression to statistically adjust for these factors, these variables must be acknowledged when interpreting the credibility and wider generalizability of the network estimates.

### Strengths and limitations

Optimizing patient outcomes and safety requires a clear understanding of whether existing medical therapies can meaningfully delay or halt AAA progression. This study represents the first NMA to comprehensively evaluate all available medical therapies investigated for AAA treatment and confirms the largely neutral findings reported in prior RCT-based analyses. Although recent observational evidence has suggested potential benefits of statins and metformin, these agents were not included in the present analysis due to the absence of RCTs assessing their effects on AAA progression or clinical outcomes.

All included trials employed intention-to-treat analyses, enhancing the generalizability of findings to routine clinical practice; however, several other limitations must be acknowledged. We express AAA growth as mm/year to standardize the growth rate, however, growth is often non-linear and may not reflect true biological progression. A further inherent limitation due to available data is the pooling of cumulative binary outcomes, such as referral to surgery and overall mortality, where using ORs does not account for differential exposure times across trials. While these are intrinsically time-to-event endpoints, the lack of available hazard ratios or patient-level time-to-event data across the included aggregate-data RCTs necessitated the use of ORs as a pragmatic alternative, which introduces potential bias. We acknowledge that without patient-level baseline diameter measurements, we are forced to assume a consistent time-at-risk and therefore may obscure early versus late event timing. Additionally, censoring due to repair/death may influence this growth estimation as well. Unfortunately, the assumption of linearity and homogeneity of slope estimation is an inherent limitation to this study.

Although NMAs are designed to compare multiple interventions, no therapy demonstrated statistical superiority, precluding meaningful treatment ranking and limiting any inference regarding comparative efficacy. Consequently, the *P*-score estimates do not imply clinical superiority, and therefore our findings reinforce that there is a current absence of convincing RCT evidence supporting medical therapy for subthreshold AAAs. Furthermore, the variable and short follow-up durations of the included trials—2 years or less in all cases—are insufficient to capture long-term disease modification, with follow-up periods of at least 5 years likely required to accrue adequate event rates and statistical power.

Collectively, these limitations underscore the ongoing uncertainty surrounding the role of medical therapy in subthreshold AAA management and highlight the need for well-designed, adequately powered, long-term trials.

### Future directions

Future AAA trials should prioritize clinically meaningful endpoints using longitudinal study designs, such as time-to-event analyses or composite outcomes including AAA surgical repair and rates of rupture, which are the principal events that medical therapies seek to delay or prevent. These approaches would enhance the robustness and clinical relevance of primary outcome assessment, as such events can be reliably ascertained through hospital record verification. However, trials powered to detect moderate treatment effects using these endpoints require substantial sample sizes, with approximately 600 events and follow-up durations of at least 5 years needed to achieve 90% statistical power. Consequently, large multicentre RCTs, potentially facilitated through established clinical research networks, will be necessary to ensure adequate patient recruitment and event accrual. Future trials should focus on promising candidate therapies, such as metformin and GLP1 inhibitors, which have demonstrated potential to influence AAA progression and may alter clinical practice. It is also plausible that medical therapies exert differential effects through distinct biological mechanisms, with treatment efficacy modulated by patient-specific factors including age, sex, and comorbidity burden. Combination therapy also needs to be investigated further. Some agents may preferentially attenuate aneurysm growth, whereas others may primarily reduce rupture risk or downstream complications, potentially accounting for the heterogeneity observed in treatment effects across studies. Furthermore, beyond treating established disease, there is a critical need to explore the prophylactic potential of these medications. Future research should include large-scale, propensity score-matched observational studies to investigate whether patients with long-term exposure to certain drugs develop aneurysms more rarely than matched controls, thereby identifying agents capable of preventing aneurysm formation rather than solely delaying growth. Finally, regardless of adjunctive medical therapy, vascular surgeons should continue to ensure regular imaging surveillance to monitor aneurysm size and growth dynamics, adhering to current guideline recommendations that integrate aneurysm diameter, growth rate, and patient life expectancy when determining the timing of surgical intervention.

## CONCLUSION

In this NMA, the evaluated medical therapies did not demonstrate a statistically significant reduction in subthreshold AAA growth rates, the need for surgical repair, or overall mortality. Rather than definitive inefficacy, these neutral findings highlight the ongoing uncertainty around modest treatment effects of medical management, limited by underpowered datasets of the currently available RCTs, with small sample sizes and short follow-up durations. To determine if medical therapies exert a modest but clinically meaningful benefit, future large-scale, methodologically robust studies must be appropriately powered to detect small but significant reductions in aneurysmal growth and need to be able to evaluate long-term, time-to-event endpoints such as surgical repair and mortality. Concurrently, future laboratory studies should prioritize the development of AAA-specific targeted therapies to maximize efficacy while mitigating treatment-limiting adverse side effects.

## FUNDING

This research did not receive any specific grant from funding agencies in the public, commercial, or not-for-profit sectors.

## CONFLICTS OF INTEREST

None declared.

## DATA AVAILABILITY

The data underlying this article will be shared on reasonable request to the corresponding author.

## Supplementary Material

ivag088_Supplementary_Data
