# Supplementary material for: The Effect of Medical Therapies for Subthreshold Abdominal Aortic Aneurysm Growth and Mortality: A Network Meta-Analysis of Randomized Controlled Trials
Source: Interdiscip Cardiovasc Thorac Surg. 2026 Mar 24;41(4):ivag088. doi: 10.1093/icvts/ivag088 (PMC13105840; doi:10.1093/icvts/ivag088)
Supplement: ivag088_Supplementary_Data [file ivag088_supplementary_data.zip › Supplement table 1.docx]

**Table 2:** Network meta-analysis of abdominal aortic aneurysm growth rate.

| Propranolol |  |  |  |  |  |  |
| --- | --- | --- | --- | --- | --- | --- |
| -0.01 (-0.68,0.65) | Telmisartan |  |  |  |  |  |
| -0.12 (-0.54,0.30) | -0.10 (-0.77,0.56) | Antibiotic |  |  |  |  |
| -0.11 (-0.42,0.19) | -0.10 (-0.70,0.50) | 0.00 (-0.29,0.30) | Placebo |  |  |  |
| -0.12 (-0.52,0.28) | -0.11 (-0.76,0.54) | -0.00 (-0.40,0.39) | -0.01 (-0.27,0.25) | ACE inhibitor |  |  |
| -0.21 (-1.15,0.72) | -0.20 (-1.26,0.86) | -0.10 (-1.02,0.83) | -0.10 (-0.98,0.78) | -0.09 (-1.01,0.83) | Ticagrelor |  |
| -0.78 (-1.57,0.00) | -0.77 (-1.71,0.17) | -0.67 (-1.45,0.12) | -0.67 (-1.40,0.06) | -0.66 (-1.44,0.11) | -0.57 (-1.71,0.57) | Pemirolast |

*The cells contain the mean difference (MD, 95% confidence interval) of the treatment on the left compared to the treatment on the right.
