# Supplementary material for: The Effect of Medical Therapies for Subthreshold Abdominal Aortic Aneurysm Growth and Mortality: A Network Meta-Analysis of Randomized Controlled Trials
Source: Interdiscip Cardiovasc Thorac Surg. 2026 Mar 24;41(4):ivag088. doi: 10.1093/icvts/ivag088 (PMC13105840; doi:10.1093/icvts/ivag088)
Supplement: ivag088_Supplementary_Data [file ivag088_supplementary_data.zip › Supplement table 4.docx]

**Table 5:** Network meta-analysis of discontinuation due to adverse events.

| Placebo |  |  |  |  |
| --- | --- | --- | --- | --- |
| 0.43 (0.16, 1.14) | ACE inhibitor |  |  |  |
| 0.31 (0.07, 1.41) | 0.73 (0.12, 4.43) | Antibiotic |  |  |
| **0.32 (0.14, 0.74)** | 0.75 (0.20, 2.73) | 1.02 (0.18, 5.76) | Propranolol |  |
| **0.20 (0.04, 0.89)** | 0.46 (0.08, 2.80) | 0.63 (0.07, 5.33) | 0.62 (0.11, 3.50) | Ticagrelor |

^§^The cells contain the odds ratio (OR, 95% confidence interval) of the treatment on the left compared to the treatment on the right. Bolded values are statistically significant.
