# Supplementary material for: The Effect of Medical Therapies for Subthreshold Abdominal Aortic Aneurysm Growth and Mortality: A Network Meta-Analysis of Randomized Controlled Trials
Source: Interdiscip Cardiovasc Thorac Surg. 2026 Mar 24;41(4):ivag088. doi: 10.1093/icvts/ivag088 (PMC13105840; doi:10.1093/icvts/ivag088)
Supplement: ivag088_Supplementary_Data [file ivag088_supplementary_data.zip › Supplement table 3.docx]

**Table 4:** Network meta-analysis of mortality

| Telmisartan |  |  |  |  |
| --- | --- | --- | --- | --- |
| 0.42 (0.07, 2.67) | Antibiotic |  |  |  |
| 0.40 (0.05, 3.22) | 0.96 (0.20, 4.57) | ACE inhibitor |  |  |
| 0.31 (0.06, 1.56) | 0.73 (0.30, 1.76) | 0.76 (0.21, 2.79) | Placebo |  |
| 0.27 (0.05, 1.44) | 0.65 (0.25, 1.68) | 0.68 (0.18, 2.61) | 0.88 (0.61, 1.29) | Propranolol |

^‡^The cells contain the odds ratio (OR, 95% confidence interval) of the treatment on the left compared to the treatment on the right. Bolded values are statistically significant.
