# Supplementary material for: The Effect of Medical Therapies for Subthreshold Abdominal Aortic Aneurysm Growth and Mortality: A Network Meta-Analysis of Randomized Controlled Trials
Source: Interdiscip Cardiovasc Thorac Surg. 2026 Mar 24;41(4):ivag088. doi: 10.1093/icvts/ivag088 (PMC13105840; doi:10.1093/icvts/ivag088)
Supplement: ivag088_Supplementary_Data [file ivag088_supplementary_data.zip › Supplement table 2.docx]

**Table 3:** Network meta-analysis of referral to aneurysm surgery.

| Propranolol |  |  |  |
| --- | --- | --- | --- |
| 0.77 (0.46, 1.31) | Antibiotic |  |  |
| 0.74 (0.52, 1.05) | 0.95 (0.64, 1.40) | Placebo |  |
| 0.24 (0.05, 1.28) | 0.31 (0.06, 1.67) | 0.33 (0.06, 1.68) | Pemirolast |

^†^The cells contain the odds ratio (OR, 95% confidence interval) of the treatment on the left compared to the treatment on the right.
